# Monitoring *Rhodnius neglectus* (Lent, 1954) populations’ susceptibility to insecticide used in controlling actions in urban areas northwest of São Paulo state

**DOI:** 10.1590/0037-8682-0553-2021

**Published:** 2022-02-25

**Authors:** Rubens Antonio da Silva, Lis Adriana Maldonado, Grasielle Caldas D’Ávila Pessoa, Liléia Diotaiuti

**Affiliations:** 1 Secretaria de Estado da Saúde de São Paulo, Superintendência de Controle de Endemias, Laboratório Especializado de Mogi Guaçu: Triatomíneos, São Paulo, SP, Brasil.; 2 Universidade Federal de Minas Gerais, Departamento de Parasitologia-ICB, Laboratório de Fisiologia de Insetos Hematófagos, Belo Horizonte, MG, Brasil.; 3 Fundação Oswaldo Cruz, Instituto René Rachou, Grupo de Pesquisa Triatomíneos, Belo Horizonte, MG, Brasil.

**Keywords:** Insecticide resistance, Rhodnius neglectus, Deltamethrin, Chagas disease, Triatominae control

## Abstract

**Background:**

Chagas disease (CD) is caused by the flagellate protozoan *Trypanosoma cruzi* and can be carried by different species of triatomines, including *Rhodnius neglectus*, which is wild, well distributed in Brazil, and has formed colonies in palm trees located in urban areas of municipalities in the state of São Paulo. Chemical control has been routinely used to reduce population density, but each year, there has been an increase in species dispersion and density. This study aimed to evaluate the susceptibility of insects to insecticides used in control.

**Methods:**

The reference population was collected from Araçatuba municipality, Nilce Maia. Dilutions of deltamethrin were prepared and applied to the back of the first-stage nymphs, which were biologically synchronized. The control group received pure acetone only. Mortality was assessed after 72 h.

**Results:**

The mortality rate with respect to diagnostic dose was 100%. The susceptibility profile observed for this population showed RR_50_ ranging from 1.76 to 3.632.

**Conclusions:**

The populations were susceptible to the insecticides tested. It is possible that the insecticide residual effect on this ecotope has decreased the lifespan, and controlling failures may be the cause of recolonization in this environment.

## INTRODUCTION

Chagas disease (CD) is caused by *Trypanosoma cruzi*, a flagellate protozoan. The infection occurs by contact with contaminated excrement of this triatomine vector, a hematophagous insect, in all of its developmental phases. Infection may also occur by intake of contaminated food, vertical transmission, blood transfusion, and organ transplant^1^.

CD is endemic to 21 Latin American countries and affects approximately 6-7 million people worldwide^2^. Recently, efforts have been made to reduce the occurrence of this infection in Latin America, but high migration rates of individuals from this region to other places have enabled DCs to spread to non-endemic countries, thereby making CD a global health issue^3^.

São Paulo state was the pioneer in controlling the main vector species, *Triatoma infestans*, and the measures adopted have become a model for other states in Brazil and South American countries^4^ ^,^ ^5^. Because of successful control measures in the domiciliary environment of *T. infestans*, entomological surveillance has focused on controlling native species that are usually found in the peridomiciliary environment^6^.

A total of 18 genera and 154 species of triatomine are known^7^. In Brazil, 64 species have been registered, 13 of these in São Paulo state, including *Rhodnius neglectus (*Lent 1954), which is mainly found in Ribeirão Preto, São José do Rio Preto, and Araçatuba. *R. neglectus* is a wild species, and its natural ecotope consists of palm trees, where it finds shelter and food. However, recent studies have reported that this species is also found in urban areas, domiciles, and peridomiciles^8^ ^,^ ^9^.

Palm trees belonging to the genus *Acrocomia* infested by *R. neglectus* feeding on *Pionnus maximiliani*, which make their nests in these palm trees, were detected in urban areas in Araçatuba, Birigui, Guararapes, and Piacatu municipalities, located in the northwest of São Paulo state^9^. Palm trees are used in landscaping design of urban areas in many cities of the state because they present low maintenance costs and are visually pleasant in the environment. The cities analyzed have a significant quantity of palm trees in their public squares and streets, thereby generating a favorable environment for these triatomines.

Controlling actions employed for palm trees located in urban areas consist of removing leaves and dry bunches, as well as chemical control by applying pyrethroid insecticide^9^. Despite this, every year, there is an increase in the dispersion and infestation of palm trees^10^. In cases of re-infestation after pyrethroid insecticide application, recolonization may occur due to insects that survived the spraying process, operational failures when applying the insecticide, or insect resistance to the product^11^.

Susceptibility to insecticide resistance has already been studied for different species, but not for *R. neglectus*. Due to infestation persistence observed in these municipalities, where chemical control has been systemically employed for over 15 years, it is necessary to assess insect susceptibility to the insecticide used in controlling measures aiming to verify its efficacy, therefore guiding actions carried out by the teams involved in such actions.

## METHODS

### Triatomines for Assessment

Samples were obtained from Araçatuba, Birigui, Guararapes, and Piacatu municipalities, all of which belong to the Araçatuba administrative region, located northwest of São Paulo state (Table 1). The insects were collected from the urban areas of these municipalities from palm trees that were regularly treated with pyrethroid insecticides provided by the Brazilian Ministry of Health under systematic applications^12^.

**TABLE 1:** Triatomine populations assessed in insecticide susceptibility test, by municipality and location. Araçatuba region, 2020.

Municipalities	Name of Location	Georeference	Insects
Araçatuba	João Arruda Brasil	-21.19291 -50.44186	300
	Alcides Chagas	-21.19555 -50.42841	210
	Escola Nilce Maia	-21.20652 -50.45417	270
Birigui	Pedro de Toledo	-21.28772 -50.34005	240
	James Mellor	-21.29140 -50.34022	240
Guararapes	Princesa Isabel	-21.24231 -50.64913	270
	Eurídes Amaral	-21.24397 -50.65012	270
	Gaudêncio José Pereira	-21.24214 -50.65025	240
	Rachel Caldas	-21.24389 -50.64647	240
	Praça Central	-21.25428 -50.64396	270
	Praça Mohamed	-21.25802 -50.64618	270
Piacatu	José Benetti	-21.58903 -50.59716	240
	Câmara Municipal	-21.58701 -50.59553	240

The insects were manually collected during the investigation procedure in the palm trees by municipal teams, kept in pots for transportation, and sent to the laboratory, where they were identified using a dichotomous key for triatomine species and classified according to their evolutionary phase^13^.

At the laboratory, they were kept in a breeding room with a constant temperature of 25 °C +3, an air relative humidity of approximately 75% +3, and a photoperiod of 12 h. The insects remained in crystallizers measuring 10 cm (height) x 23.5 cm (diameter), lined with filtering paper to absorb excrement and humidity, with a hive-shaped 2 mm thick pressed cardboard sheet support and covered with thick dark cotton to protect them from light. The insects were fed through an artificial method with the rustic chicken, *Rhodia sp.*, on a weekly basis.

### Reference Population

A reference population of *R. neglectus* was selected, in which laboratory breeding started on 12/01/2004 and the insects were from Araçatuba municipality, Nilce Maia location. Specifically, it was necessary to identify the relevant diagnostic dose under which mortality of 50% of the population was observed (LD_50_). The criteria for selecting the reference population followed the World Health Organization guidelines^14^. 

Tests were performed to identify the LD_50_ and LD_99_ in this population. After reading the nymph death for the eight dilution rates used, the numbers obtained were organized in a reading database for the Polo Plus^®^ software. RR_50_ and RR_99_ were obtained by dividing the LD_50_ of the field population by the LD_50_ of the susceptible population, with correspondence to calculate the RR_99_. A confidence interval of 95% (CI 95%) of each population was also calculated for each population.

### Insecticide Susceptibility Test

The methodology used to monitor insect resistance in the laboratory was standardized by Pessoa (2016)^15^ and defines insect generation, nymph age, insecticide application *locus*, and the ideal diagnostic dose for each species. The insects collected were kept in a quarantine after arriving at the laboratory and, at the end of this period, eggs were removed and the first stage nymphs’ birth was synchronized in order to perform the experiment.

The pyrethroid deltamethrin (99.1% purity) was supplied by Bayer. A total volume of 0.1 *u*L was applied on the back of the nymph on the fifth day after birth. The control groups received only pure acetone on their backs. Readings were performed 72 h after application, considering the normal, intoxicated, and dead nymphs. For each dilution, 30 insects were used at the first stage, with at least eight doses necessary to determine the mortality curve. Three groups were formed, with 10 insects in each group, and the tests were carried out on different days.

Mortality data were analyzed using the Basic Probit Analysis program to estimate the slope and lethal dose (LD) in nanograms of active ingredients per treated nymph. The susceptibility classification was in accordance with the Pan American Health Organization^14^. After defining the *R. neglectus* susceptibility baseline for the reference population, 30 nymphs from each field population were subjected to a diagnostic dose of 1xLD_99._ The survival of at least two insects in three repetitions was interpreted as a resistance indicator. 

## RESULTS

The susceptibility to deltamethrin in the reference population was 0.025 ng/treated nymphs at LD_50_. At this diagnostic dose, 100% mortality was observed in all populations. Regarding *R. neglectus* samples from field populations, variations between 0.0440 and 0.0908 ng/treated nymphs were observed (Table 2). Populations from Birigui (James Mellor and Pedro de Toledo) and Guararapes (Praça Mohamed and Princesa Isabel) presented lower slopes than the reference population, indicating less homogeneous populations with a higher resistance selection likelihood. 

**TABLE 2:** Result of mortality of first stage triatomine nymphs of *Rhodnius neglectus* after topical application of deltamethrin in a reference population and in samples from different locations. Araçatuba region, 2020.

Municipalities	Locations	LD_50_ (IC 95%)	LD_99_ (IC 95%)	RR_50_**	Slope
Araçatuba	Nilce Maia - LRS*	0.0250 (0.0020 - 0.0320)	0.1960 (0.0108 - 0.3310)	-	2.520+-0.428
Araçatuba	Alcides Chagas	0.0823 (0.0691 - 0.1014)	0.6096 (0.3453 - 1.7103)	3.292	2.675+-0.436
	Arruda Brasil	0.0633 (0.0502 - 0.0819)	0.6209 (0.3293 - 2.1666)	2.532	3.346+-0.294
	Escola Nilce Maia	0.0741 (0.0556 - 0.0977)	0.3904 (0.2178 -2.0769)	2.964	3.223+-0.437
Birigui	James Mellor	0.0484 (0.0349 - 0.0733)	0.6723 (0.2757 - 5.8711)	1.936	2.035+-0.294
	Pedro de Toledo	0.0440 (0.0361 - 0.0528)	0.4049 (0.2543 - 0.8754)	1.76	2.413+-0.320
Guararapes	Eurides Amaral	0.0775 (0.0680 - 0.0903)	0.4439 (0.2863 - 0.9319)	3.1	3.088+-0.446
	Gaudêncio José	0.0871 (0.0738 - 0.1047)	0.6786 (0.4047 - 1.7420)	3.484	2.609+-0.400
	Praça Central	0.0739 (0.0590 - 0.0921)	0.4040 (0.2422 - 1.2799)	2.956	3.154+-0.409
	Praça Mohamed	0.0674 (0.0557 - 0.0817)	0.8291 (0.4623 - 2.3296)	2.696	2.134+-0.311
	Princesa Isabel	0.0845 (0.0707 - 0.1049)	0.9821 (0.5095 - 3.4704)	3.38	2.184+-0.359
	Raquel Caldas	0.0681 (0.0580 - 0.0790)	0.4120 (0.2811 - 0.7801)	2.724	2.976+-0.399
Piacatu	Câmara Municipal	0.0908 (0.0767 - 0.1149)	0.3839(0.2406 - 1.0276)	3.632	3.716+-0.522
	José Benetti	0.0754 (0.0661 - 0.0853)	0.3151 (0.2348 - 0.5057)	3.016	3.744+-0.472

*Reference population susceptibility, **Resistance ratio 50% ng a.i./treated nymph (nanogram active ingredient / treated nymph.

Values obtained for RR_50_ in the field populations were significantly different from those in the reference population, and there was no superposition of confidence interval limits at 95%.

## DISCUSSION

Insecticide resistance can be understood as a decrease in mortality observed in a population that has undergone constant exposure to chemical products used for extermination^11^. In the case of triatomines, such resistance is regarded as rare and unlikely, mainly because of the life cycle of these insects, which hinders the selection of resistant individuals^11^. However, previous studies have demonstrated cases of triatomine populations resistant to numerous active substances in different regions of America^16^ ^-^ ^18^.

Triatomine resistance to pyrethroids associated with ineffective field treatment has been reported in *R. prolixus* in Venezuela and in *Triatoma infestans* in Brazil, Argentina, and Bolivia^19^. After these findings, studies aimed at verifying susceptibility in Brazilian populations of *Triatoma infestans*, *T. sordida*, and *T. brasiliensis* were carried out and have found, in some situations, resistance to the deltamethrin pyrethroid insecticide^18^ ^,^ ^20^ ^-^ ^24^.

The first report of pyrethroid insecticide resistance in triatomines of the genus *Rhodnius* was observed in populations of *R. prolixus* species in Venezuela using dieldrin^25^. There are no reports of studies conducted in Brazil to verify insecticide resistance susceptibility in populations of *Rhodnius* genus, which may be due to the fact that most species from these genus do not inhabit homes, therefore posing secondary risk to human beings.

In São Paulo state, *R. neglectus* is the second most collected species and, currently, most samples are from urban areas where there is a superposition of controlling actions aimed at arboviruses and visceral leishmaniasis^26^. To chemically control vectors, the Brazilian Ministry of Health provides states and municipalities with alpha-cypermethrin, a pyrethroid insecticide, as this insecticide has a low residual effect, does not remain on treated surfaces for long periods, and is subjected to weather changes, which may impair its useful life^17^. This insecticide is also widely used because of its low toxicity in mammals and because it does not persist in the environment^27^.

Chemical control employed frequently to decrease infestation of this species in urban areas has not presented satisfactory results, which may lead to metabolic changes in the insects, resulting in resistance to the insecticides applied to control them. However, results from bioassays for this triatomine species did not indicate resistance; yet, there is evidence concerning the need for management strategies to maintain the insecticide lifespan, but it is necessary to continuously test insect resistance in these insect populations. 

Values obtained for RR_50_ in this assessment, and considering that values higher or equal to 5 are parameters to characterize insect resistance to the insecticide, indicated that controlling actions may proceed with the same insecticide; however, new management methodologies must be considered for the palm trees. Mechanical control of this vector species in this ecotope must also be prioritized, since it is a fact that this insect currently inhabits an urban area in São Paulo state and its widening distribution will bring new challenges.

Pessoa et al. (2016)^15^ defined a reference population for *R. neglectus* in which the LD_50_ value was 0.001 ng i. a/ninfa. If this value is considered, all populations tested in this assessment would be classified as resistant, indicating that it may be necessary to locally standardize the species under study. Obara et al. (2011)^20^ and Pessoa et al. (2014)^21^ also found such a need when working with *T. sordida*.

It is important to note that the reference population in this study was collected from the same area where samples used in this assessment were collected, that is, Araçatuba municipality, Nilce Maria location. However, spaying insecticide on the palm trees to control insect populations was not a technical standard at the time. As other vectors are also found in this area, pyrethroids are frequently used in controlling actions, which must be considered for the reference population, as they may show signs of resistance.

The fact that the slope was the same or higher than that of the reference population shows that, in most populations tested, there is little heterogeneity among them. For populations with lower slopes, resistance can be observed, which justifies the follow-up. Molecular studies have indicated less genetic diversity in areas treated with chemical treatment^28^. It is important to highlight that the mortality at the diagnostic dose was 100% in all populations. Genetic variation in populations must be considered as a factor that may directly interfere with test results^21^ ^,^ ^29^.

Finally, it is essential to analyze triatomine resistance to insecticides through studies aiming to better understand which factors may affect the control of these vectors, in order to evaluate and enhance intervention measures, if necessary. Previous studies have reported *R. neglectus* populations in palm trees located in urban areas in the Federal District of Goiás, Minas Gerais, and Mato do Grosso do Sul states^8^ ^,^ ^30^ ^,^ ^31^. It is not uncommon to find notifications to public authorities concerning these insects by inhabitants of these areas, indicating that new epidemiological settings may occur. 

Recolonization of this species in the environment may be related to behavioral factors and to the fact that they are also associated with birds, which facilitate its wide distribution in municipalities within the São Paulo state^9^ ^,^ ^32^. Therefore, studies using field and laboratory insecticides simultaneously must be carried out to obtain results that are closer to local reality.
